# EBHI-Seg: A novel enteroscope biopsy histopathological hematoxylin and eosin image dataset for image segmentation tasks

**DOI:** 10.3389/fmed.2023.1114673

**Published:** 2023-01-24

**Authors:** Liyu Shi, Xiaoyan Li, Weiming Hu, Haoyuan Chen, Jing Chen, Zizhen Fan, Minghe Gao, Yujie Jing, Guotao Lu, Deguo Ma, Zhiyu Ma, Qingtao Meng, Dechao Tang, Hongzan Sun, Marcin Grzegorzek, Shouliang Qi, Yueyang Teng, Chen Li

**Affiliations:** ^1^Microscopic Image and Medical Image Analysis Group, College of Medicine and Biological Information Engineering, Northeastern University, Shenyang, China; ^2^Department of Pathology, Cancer Hospital of China Medical University, Liaoning Cancer Hospital and Institute, Shengyang, China; ^3^Shengjing Hospital, China Medical University, Shenyang, China; ^4^Institute of Medical Informatics, University of Lübeck, Lübeck, Germany; ^5^Department of Knowledge Engineering, University of Economics in Katowice, Katowice, Poland

**Keywords:** colorectal histopathology, enteroscope biopsy, image dataset, image segmentation, EBHI-Seg

## Abstract

**Background and purpose:**

Colorectal cancer is a common fatal malignancy, the fourth most common cancer in men, and the third most common cancer in women worldwide. Timely detection of cancer in its early stages is essential for treating the disease. Currently, there is a lack of datasets for histopathological image segmentation of colorectal cancer, which often hampers the assessment accuracy when computer technology is used to aid in diagnosis.

**Methods:**

This present study provided a new publicly available *Enteroscope Biopsy Histopathological Hematoxylin and Eosin Image Dataset for Image Segmentation Tasks* (EBHI-Seg). To demonstrate the validity and extensiveness of EBHI-Seg, the experimental results for EBHI-Seg are evaluated using classical machine learning methods and deep learning methods.

**Results:**

The experimental results showed that deep learning methods had a better image segmentation performance when utilizing EBHI-Seg. The maximum accuracy of the Dice evaluation metric for the classical machine learning method is 0.948, while the Dice evaluation metric for the deep learning method is 0.965.

**Conclusion:**

This publicly available dataset contained 4,456 images of six types of tumor differentiation stages and the corresponding ground truth images. The dataset can provide researchers with new segmentation algorithms for medical diagnosis of colorectal cancer, which can be used in the clinical setting to help doctors and patients. EBHI-Seg is publicly available at: https://figshare.com/articles/dataset/EBHI-SEG/21540159/1.

## 1. Introduction

Colon cancer is a common deadly malignant tumor, the fourth most common cancer in men, and the third most common cancer in women worldwide. Colon cancer is responsible for 10% of all cancer cases (1). According to prior research, colon and rectal tumors share many of the same or similar characteristics. Hence, they are often classified collectively (2). The present study categorized rectal and colon cancers into one colorectal cancer category (3). Histopathological examination of the intestinal tract is both the gold standard for the diagnosis of colorectal cancer and a prerequisite for disease treatment (4).

The advantage of using the intestinal biopsy method to remove a part of the intestinal tissue for histopathological analysis, which is used to determine the true status of the patient, is that it considerably reduces damage to the body and rapid wound healing (5). The histopathology sample is then sectioned and processed with Hematoxylin and Eosin (H&E). Treatment with H&E is a common approach when staining tissue sections to show the inclusions between the nucleus and cytoplasm and highlight the fine structures between tissues (6, 7). When a pathologist performs an examination of the colon, they first examine the histopathological sections for eligibility and find the location of the lesion. The pathology sections are then examined and diagnosed using a low magnification microscope. If finer structures need to be observed, the microscope is adjusted to use high magnification for further analysis. However, the following problems usually exist in the diagnostic process: the diagnostic results become more subjective and varied due to different doctors reasons; doctors can easily overlook some information in the presence of a large amount of test data; it is difficult to analyze large amounts of previously collected data (8). Therefore, it is a necessary to address these issues effectively.

With the development and popularization of computer-aided diagnosis (CAD), the pathological sections of each case can be accurately and efficiently examined with the help of computers (9). Now, CAD is widely used in many biomedical image analysis tasks, such as microorganism image analysis (10–18), COVID-19 image analysis (19), histopatholgical image analysis (20–27), cytopathological image analysis (28–31) and sperm video analysis (32, 33). Therefore, the application of computer vision technology for colorectal cancer CAD provides a new direction in this research field (34).

One of the fundamental tasks of CAD is the aspect of image segmentation, the results of which can be used as key evidence in the pathologists' diagnostic processes. Along with the rapid development of medical image segmentation methodology, there is a wide demand for its application to identify benign and malignant tumors, tumor differentiation stages, and other related fields (35). Therefore, a multi-class image segmentation method is needed to obtain high segmentation accuracy and good robustness (36).

The present study presents a novel *Enteroscope Biopsy Histopathological H*&*E Image Dataset for Image Segmentation Tasks* (EBHI-Seg), which contains 4456 electron microscopic images of histopathological colorectal cancer sections that encompass six tumor differentiation stages: normal, polyp, low-grade intraepithelial neoplasia, high-grade intraepithelial neoplasia, serrated adenoma, and adenocarcinoma. The segmentation coefficients and evaluation metrics are obtained by segmenting the images of this dataset using different classical machine learning methods and novel deep learning methods.

## 2. Related work

The present study analyzed and compared the existing colorectal cancer biopsy dataset and provided an in-depth exploration of the currently known research findings. The limitations of the presently available colorectal cancer dataset were also pointed out.

The following conclusions were obtained in the course of the study. For existing datasets, the data types can be grouped into two major categories: Multi and Dual Categorization datasets. Multi Categorization datasets contain tissue types at all stages from Normal to Neoplastic. In Trivizakis et al. (37), a dataset called “Collection of textures in colorectal cancer histology” is described. It includes 5,000 patches of size 74 × 74 μm and contains seven categories. However, because there were only 10 images, it is too small for a data sample and lacked generalization capability. In Chen et al. (23), a dataset called “NCT-CRC-HE-100K” is proposed. This is a set of 100,000 non-overlapping image patches of histological human colorectal cancer (CRC) and normal tissue samples stained with (H&E) that was presented by the National Center for Tumor Diseases (NCT). These image patches are from nine different tissues with an image size of 224 × 224 pixels. The nine tissue categories are adipose, background, debris, lymphocytes, mucus, smooth muscle, normal colon mucosa, cancer-associated stroma, and colorectal adenocarcinoma epithelium. This dataset is publicly available and commonly used. However, because the image sizes are all 224 × 224 pixels, the dataset underperformed in some global details that need to be observed in individual categories. Two datasets are utilized in Oliveira et al. (38): one containing colonic H&E-stained biopsy sections (CRC dataset) and the other consisting of prostate cancer H&E-stained biopsy sections (PCa dataset). The CRC dataset contains 1,133 colorectal biopsy and polypectomy slides grouped into three categories and labeled as non-neoplastic, low-grade and high-grade lesions. In Kausar et al. (39), a dataset named “MICCAI 2016 gland segmentation challenge dataset (GlaS)” is used. This dataset contained 165 microscopic images of H&E-stained colon glandular tissue samples, including 85 training and 80 test datasets. Each dataset is grouped into two parts: benign and malignant tumors. The image size is 775 × 522 pixels. Since this dataset has only two types of data and the number of data is too little, so that it performs poorly on some multi-type training.

Dual Categorization datasets usually contain only two types of tissue types: Normal and Neoplastic. In Wei et al. (40), a dataset named “FFPE” is proposed. This dataset obtained its images by extracting 328 Formalin-fixed Paraffin-embedded (FFPE) whole-slide images of colorectal polyps classified into two categories of : hyperplastic polyps (HPs) and sessile serrated adenomas (SSAs). This dataset contained 3,125 images with an image size of 224 × 224 pixels and is small in type and number. In Bilal et al. (41), two datasets named “UHCW” and “TCGA” are proposed. The first dataset is a colorectal cancer biopsy sequence developed at the University Hospital of Coventry and Warwickshire (UHCW) for internal validation of the rectal biopsy trial. The second dataset is the Cancer Genome Atlas (TCGA) for external validation of the trial. This dataset is commonly used as a publicly available cancer dataset and stores genomic data for more than 20 types of cancers. The two dataset types are grouped into two categories: Normal and Neoplastic. The first dataset contains 4,292 slices, and the second dataset contained 731 slices with an image size of 224 × 224 pixels.

All of the information for the existing datasets is summarized in Table 1. The issues associated with the dataset mentioned above included fewer data types, small amount of data, inaccurate dataset ground truth, etc. The current study required an open-source multi-type colonoscopy biopsy image dataset.

**Table 1:** A dataset for the pathological classification of colorectal cancer.

	**Dataset name**	**Categorization**	**Amount**	**Size**	**Year**
Multi categorization	Collection of textures in colorectal cancer histology	Lymphoid follicles, mucosal glands, debris, adipose, tumor epithelium simple stroma, complex stroma, background patches with no tissue	5,000	74 × 74 μm (0.495 micrometer per pixel)	2016
	HE-NCT-CRC-100K	MUS, NORM, STR, TUM ADI, BACK, DEB, LYM, MUC	100,000	224 × 224 pixels	2016
	MICCAI'16 gland seg-mentation challenge dataset	Benign tumors, malignant tumors	85	775 × 522 pixels	2017
	CRC dataset	Non-neoplastic, low-grade, high-grade lesions	1,133	512 × 512 pixels	2021
Dual categorization	FFPE	HPs, SSAs	3,152	224 × 224 pixels	2021
	The Cancer Genome Atlas dataset	Normal, Neoplastic	731	224 × 224 pixels	2021
	University Hospitals Coventry and Warwick-shire dataset	Normal, Neoplastic	4,292	224 × 224 pixels	2021

## 3. Basic information for EBHI-Seg

### 3.1. Dataset overview

The dataset in the present study contained 4,456 histopathology images, including 2,228 histopathology section images and 2,228 ground truth images. These include normal (76 images and 76 ground truth images), polyp (474 images and 474 ground truth images), low-grade intraepithelial neoplasia (639 images and 639 ground truth images), high-grade intraepithelial neoplasia (186 images and 186 ground truth images), serrated adenoma (58 images and 58 ground truth images), and adenocarcinoma (795 images and 795 ground truth images). The basic information for the dataset is described in detail below. EBHI-Seg is publicly available at: https://figshare.com/articles/dataset/EBHI-SEG/21540159/1.

In the present paper, H&E-treated histopathological sections of colon tissues are used as data for evaluating image segmentation. The dataset is obtained from two histopathologists at the Cancer Hospital of China Medical University [proved by “Research Project Ethics Certification” (No. 202229)]. It is prepared by 12 biomedical researchers according to the following rules: Firstly, if there is only one differentiation stage in the image and the rest of the image is intact, then the differentiation stage became the image label; Secondly, if there is more than one differentiation stage in the image, then the most obvious differentiation is selected as the image label; In general, the most severe and prominent differentiation in the image was used as the image label.

Intestinal biopsy was used as the sampling method in this dataset. The magnification of the data slices is 400×, with an eyepiece magnification of 10× and an objective magnification of 40×. A Nissan Olympus microscope and NewUsbCamera acquisition software are used. The image input size is 224 × 224 pixels, and the format is *.png. The data are grouped into five types described in detail in Section 2.2.

### 3.2. Data type description

#### 3.2.1. Normal

**Figure 1:**
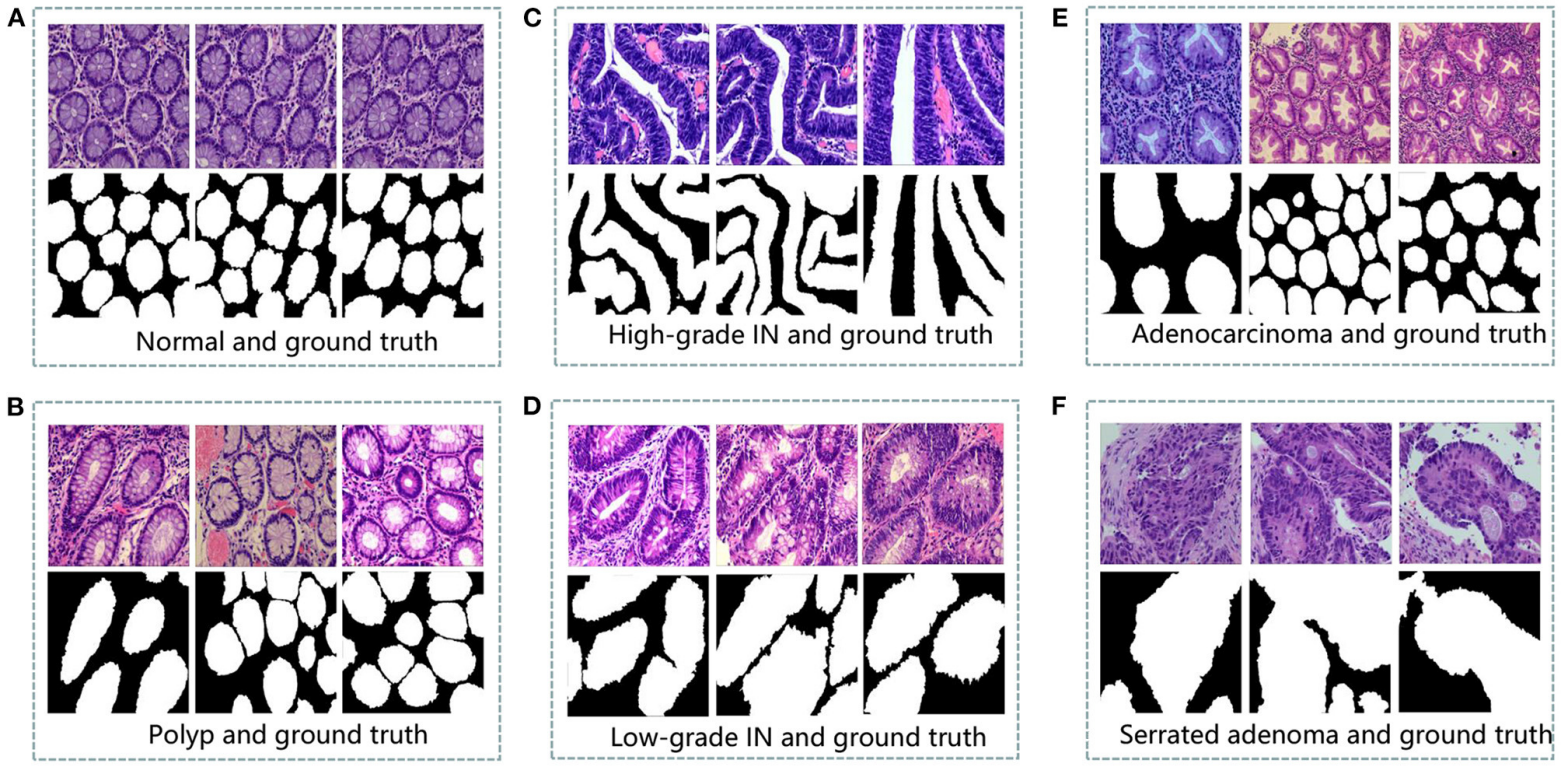
An example of histopathological images database. **(A)** Normal and ground truth, **(B)** Polyp and ground truth, **(C)** High-grade Intraepithelial Neoplasia and ground truth, **(D)** Low-grade Intraepithelial Neoplasia and ground truth, **(E)** Adenocarcinoma and ground truth, and **(F)** Serrated adenoma and ground truth.

Colorectal tissue sections of the standard category are made-up of consistently ordered tubular structures and that does not appear infected when viewed under a light microscope (42). Section images with the corresponding ground truth images are shown in Figure 1A.

#### 3.2.2. Polyp

Colorectal polyps are similar in shape to the structures in the normal category, but have a completely different histological structure. A polyp is a redundant mass that grows on the surface of the body's cells. Modern medicine usually refers to polyps as unwanted growths on the mucosal surface of the body (43). The pathological section of the polyp category also has an intact luminal structure with essentially no nuclear division of the cells. Only the atomic mass is slightly higher than that in the normal category. The polyp category and corresponding ground truth images are shown in Figure 1B.

#### 3.2.3. Intraepithelial neoplasia

Intraepithelial neoplasia (IN) is the most critical precancerous lesion. Compared to the normal category, its histological images show increased branching of adenoid structures, dense arrangement, and different luminal sizes and shapes. In terms of cellular morphology, the nuclei are enlarged and vary in size, while nuclear division increases (44). The standard Padova classification currently classifies intraepithelial neoplasia into low-grade and high-grade INs. High-grade IN demonstrate more pronounced structural changes in the lumen and nuclear enlargement compared to low-grade IN. The images and ground truth diagrams of high-grade and low-grade INs are shown in Figures 1C, D.

#### 3.2.4. Adenocarcinoma

Adenocarcinoma is a malignant digestive tract tumor with a very irregular distribution of luminal structures. It is difficult to identify its border structures during observation, and the nuclei are significantly enlarged at this stage (45). An adenocarcinoma with its corresponding ground truth diagram is shown in Figure 1E.

#### 3.2.5. Serrated adenoma

Serrated adenomas are uncommon lesions, accounting for 1% of all colonic polyps (46). The endoscopic surface appearance of serrated adenomas is not well characterized but is thought to be similar to that of colonic adenomas with tubular or cerebral crypt openings (47). The image of a serrated adenoma with a corresponding ground truth diagram is shown in Figure 1F.

## 4. Evaluation of EBHI-Seg

### 4.1. Image segmentation evaluation metric

Six evaluation metrics are commonly used for image segmentation tasks. The Dice ratio metric is a standard metric used in medical images that is often utilized to evaluate the performance of image segmentation algorithms. It is a validation method based on spatial overlap statistics that measures the similarities between the algorithm segmentation output and ground truth (48). The Dice ratio is defined in Equation (1).


(1) $$\begin{array}{l} \text{DiceRatio}=\frac{2|\text{X}\cap \text{Y}|}{\left|\text{X}|+|\text{Y}\right|}. \end{array}$$

In Equation (1), for a segmentation task, *X* and *Y* denote the ground truth and segmentation mask prediction, respectively. The range of the calculated results is [0,1], and the larger the result the better.

The Jaccard index is a classical set similarity measure with many practical applications in image segmentation. The Jaccard index measures the similarity of a finite set of samples: the ratio between the intersection and concatenation of the segmentation results and ground truth (49). The Jaccard index is defined in Equation (2).


(2) $$\begin{array}{l} \text{JaccardIndex}=\frac{\left|\text{X}\cap \text{Y}\right|}{\left|\text{X}\cup \text{Y}\right|}. \end{array}$$

The range of the calculated results is [0,1], and the larger the result the better.

Recall and precision are the recall and precision rates, respectively. The range of the calculated results is [0,1]. A higher output indicates a better segmentation result. Recall and precision are defined in Equations (3), (4),


(3) $$\begin{array}{l} \text{Precison}=\frac{\text{TP}}{\text{TP}+\text{FP}}, \end{array}$$

(4) $$\begin{array}{l} \text{Recall}=\frac{\text{TP}}{\text{TP}+\text{FN}}, \end{array}$$

where TP, FP, TN, and FN are defined in Table 2.

**Table 2:** Confusion matrix.

**Ground truth**	**Predict mask**	
	**Positive**	**Negative**
Positive	TP	TN
Negative	FP	FN

The conformity coefficient (Confm Index) is a consistency coefficient, which is calculated by putting the binary classification result of each pixel from [−∞,1] into continuous interval [−∞,1] to calculate the ratio of the number of incorrectly segmented pixels to the number of correctly segmented pixels to measure the consistency between the segmentation result and ground truth. The conformity coefficient is defined in Equations (5), (6),


(5) $$\begin{array}{l} \text{ConfmIndex}=\left(1-\frac{\theta_{\text{AE}}}{\theta_{\text{TP}}}\right),\theta_{\text{TP}}>0, \end{array}$$

(6) $$\begin{array}{l} \text{ConfmIndex}=\text{Failure},\theta_{\text{TP}}=0, \end{array}$$

Where θ_*AE*_= θ_*FP*_+θ_*FN*_ represents all errors of the fuzzy segmentation results. θ_*TP*_ is the number of correctly classified pixels. Mathematically, ConfmIndex can be negative infinity if θ_*TP*_=0. Such a segmentation result is definitely inadequate and treated as failure without the need of any further analysis.

### 4.2. Classical machine learning methods

**Figure 2:**
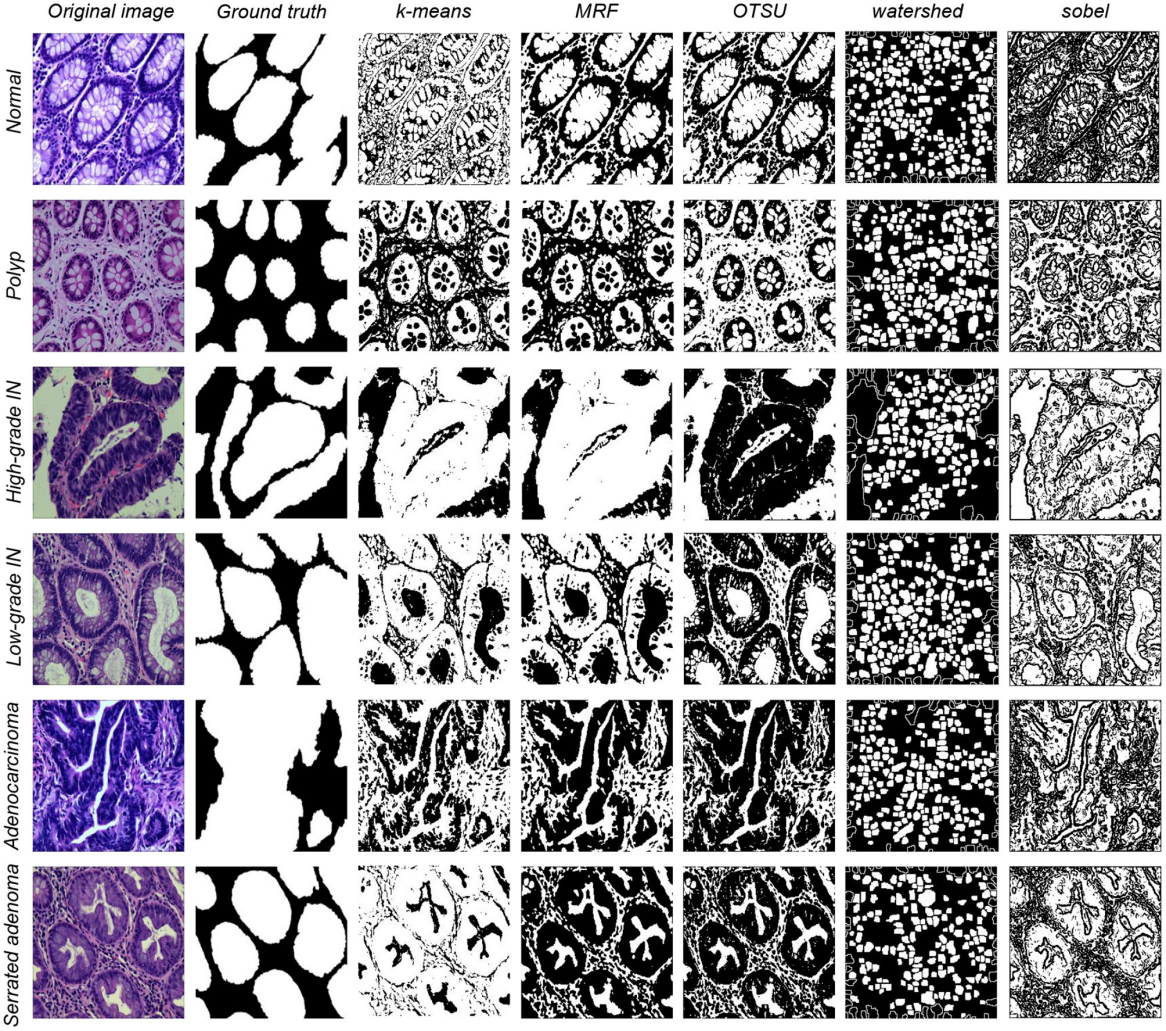
Five types of data segmentation results obtained by different classical machine learning methods.

Image segmentation is one of the most commonly used methods for classifying image pixels in decision-oriented applications (50). It groups an image into regions high in pixel similarity within each area and has a significant contrast between different regions (51). Machine learning methods for segmentation distinguish the image classes using image features. (1) *k*-means algorithm is a classical division-based clustering algorithm, where image segmentation means segmenting the image into many disjointed regions. The essence is the clustering process of pixels, and the *k*-means method is one of the simplest clustering methods (52). Image segmentation of the present study dataset is performed using the classical machine learning method described above. (2) Markov random field (MRF) is a powerful stochastic tool that models the joint probability distribution of an image based on its local spatial action (53). It can extract the texture features of the image and model the image segmentation problem. (3) OTSU algorithm is a global adaptive binarized threshold segmentation algorithm that uses the maximum inter-class variance between the image background and the target image as the selection criterion (54). The image is grouped into foreground and background parts based on its grayscale characteristics independent of the brightness and contrast. (4) Watershed algorithm is a region-based segmentation method, that takes the similarity between neighboring pixels as a reference and connects those pixels with similar spatial locations and grayscale values into a closed contour to achieve the segmentation effect (55). (5) Sobel algorithm has two operators, where one detects horizontal edges and the other detects vertical flat edges. An image is the final result of its operation. Sobel edge detection operator is a set of directional operators that can be used to perform edge detection from different directions (56). The segmentation results are shown in Figure 2.

The performance of EBHI-Seg for different machine learning methods is observed by comparing the images segmented using classical machine learning methods with the corresponding ground truth. The segmentation evaluation metrics results are shown in Table 3. The Dice ratio algorithm is a similarity measure, usually used to compare the similarity of two samples. The value of one for this metric is c onsidered to indicate the best effect, while the value of the worst impact is zero. The Table 3 shows that *k*-means has a good Dice ratio algorithm value of up to 0.650 in each category. The MRF and Sobel segmentation results also achieved a good Dice ratio algorithm value of around 0.6. In terms of image precision and recall segmentation coefficients, *k*-means is maintained at approximately 0.650 in each category. In the classical machine learning methods, *k*-means has the best segmentation results, followed by MRF and Sobel. OTSU has a general effect, while the watershed algorithm has various coefficients that are much lower than those in the above methods. Moreover, there are apparent differences in the segmentation results when using the above methods.

**Table 3:** Evaluation metrics for five different segmentation methods based on classical machine learning.

		**Dice ratio**	**JaccardIndex**	**Conformity coefficient**	**Precision**	**Recall**
Normal	*k*-means	0.648	0.488	–0.184	0.646	0.663
	MRF	0.636	0.473	–0.230	0.637	0.658
	OTSU	0.410	0.265	–2.871	0.515	0.351
	Watershed	0.461	0.300	–1.375	0.668	0.356
	Sobel	0.652	0.487	–0.102	0.763	0.579
Polyp	*k*-means	0.592	0.430	–0.528	0.546	0.663
	MRF	0.511	0.362	–2.133	0.540	0.502
	OTSU	0.400	0.259	–3.108	0.413	0.399
	Watershed	0.433	0.277	–1.675	0.551	0.362
	Sobel	0.583	0.416	–0.499	0.626	0.562
High-grade IN	*k*-means	0.626	0.478	–0.467	0.650	0.620
	MRF	0.550	0.441	–30.85	0.614	0.526
	OTSU	0.249	0.150	–12.06	0.373	0.191
	Watershed	0.472	0.309	–1.258	0.738	0.350
	Sobel	0.634	0.469	–0.200	0.728	0.577
Low-grade IN	*k*-means	0.650	0.492	–0.172	0.651	0.663
	MRF	0.554	0.404	–1.808	0.643	0.504
	OTSU	0.886	0.811	0.6998	0.832	0.979
	Watershed	0.464	0.303	–1.345	0.676	0.357
	Sobel	0.656	0.492	–0.079	0.771	0.582
Adenocarcinoma	*k*-means	0.633	0.481	–0.414	0.655	0.645
	MRF	0.554	0.404	–1.808	0.643	0.504
	OTSU	0.336	0.215	–5.211	0.454	0.282
	Watershed	0.458	0.298	–1.437	0.700	0.349
	Sobel	0.553	0.388	–0.733	0.692	0.484
Serrated adenoma	*k*-means	0.636	0.473	–0.230	0.637	0.658
	MRF	0.571	0.419	–0.898	0.656	0.547
	OTSU	0.393	0.248	–2.444	0.565	0.315
	Watershed	0.449	0.290	–1.494	0.656	0.345
	Sobel	0.698	0.541	0.7484	0.662	0.572

In summary, EBHI-Seg has significantly different results when using different classical machine learning segmentation methods. Different classical machine learning methods have an obvious differentiation according to the image segmentation evaluation metrics. Therefore, EBHI-Seg can effectively evaluate the segmentation performance of different segmentation methods.

### 4.3. Deep learning methods

**Figure 3:**
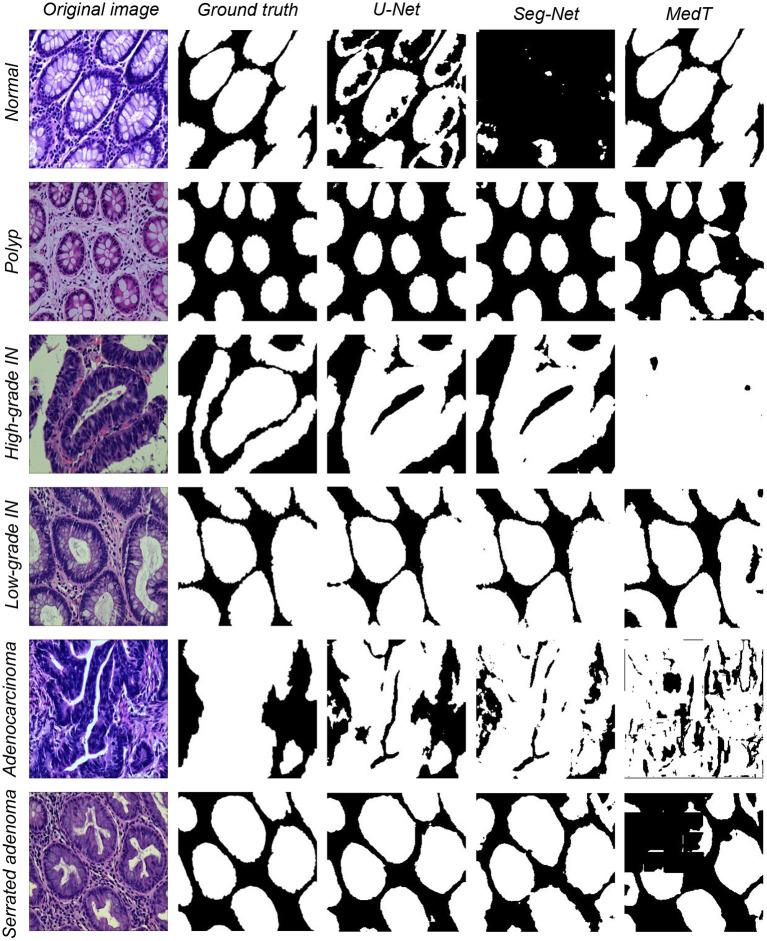
Three types of data segmentation results obtained by different deep learning methods.

Besides the classical macine learning metheds tested above, some popular deep learning methods are also tested. (1) Seg-Net is an open source project for image segmentation (57). The network is identical to the convolutional layer of VGG-16, with the removal of the fully-connected hierarchy and the addition of max-pooling indices resulting in improved boundary delineation. Seg-Net performs better in large datasets. (2) U-Net network structure was first proposed in 2015 (58) for medical imaging. U-Net is lightweight, and its simultaneous detection of local and global information is helpful for both information extraction and diagnostic results from clinical medical images. (3) MedT is a network published in 2021, which is a transformer structure that applies an attention mechanism based on medical image segmentation (59). The segmentation results are shown in Figure 3.

The segmentation effect is test on the present dataset using three deep learning models. In the experiments, each model is trained using the ratio of the training set, validation set, and test set of 4:4:2. All of the information for the existing datasets is summarized in Table 4. The model learning rate is set to 3*e*−6, epochs are set to 100, and batch-size is set to 1. The optimizer is Adam, the loss function is crossentropyloss and the activation function is ReLU. The dataset segmentation results of using three different models are shown in Figure 3. The experimental segmentation evaluation metrics are shown in Table 5. Overall, deep learning performs much better than classical machine learning methods. Among them, the evaluation indexes of the training results using the U-Net and Seg-Net models can reach 0.90 on average. The evaluation results of the MedT model are slightly worse at a level, between 0.70 and 0.80. The training time is longer for MedT and similar for U-Net and Seg-Net.

**Table 4:** Deep learning of the number of different types of training images.

	**Train**	**Test**	**Predict**
Normal	30	30	16
Polyp	190	190	94
Low-grade IN	256	256	127
High-grade IN	74	74	38
Serrated adenoma	23	23	12
Adenocarcinoma	318	318	159

**Table 5:** Evaluation metrics for three different segmentation methods based on deep learning.

		**Dice ratio**	**JaccardIndex**	**Conformity coefficient**	**Precision**	**Recall**
Normal	U-Net	0.411	0.263	–2.199	0.586	0.328
	Seg-Net	0.777	0.684	–0.607	0.895	0.758
	MedT	0.676	0.562	–0.615	0.874	0.610
Polyp	U-Net	0.965	0.308	–1.514	0.496	0.470
	Seg-Net	0.937	0.886	0.858	0.916	0.965
	MedT	0.771	0.643	0.336	0.687	0.920
High-grade IN	U-Net	0.895	0.816	0.747	0.847	0.961
	Seg-Net	0.894	0.812	0.757	0.881	0.913
	MedT	0.824	0.707	0.556	0.740	0.958
Low-grade IN	U-Net	0.911	0.849	0.773	0.879	0.953
	Seg-Net	0.924	0.864	0.826	0.883	0.977
	MedT	0.889	0.808	0.730	0.876	0.916
Adenocarcinoma	U-Net	0.887	0.808	0.718	0.850	0.950
	Seg-Net	0.865	0.775	0.646	0.792	0.977
	MedT	0.735	0.595	0.197	0.662	0.864
Serrated adenoma	U-Net	0.938	0.886	0.865	0.899	0.983
	Seg-Net	0.907	0.832	0.794	0.859	0.963
	MedT	0.670	0.509	–0.043	0.896	0.544

Based on the above results, EBHI-Seg achieved a clear differentiation using deep learning image segmentation methods. Image segmentation metrics for different deep learning methods are significantly different so that EBHI-Seg can evaluate their segmentation performance.

### 4.4. Experimental environment

This section presents the hardware configuration data required for this experiment as well as the software version.

Processor: Intel Core i7-8700 @ 3.20GHz Six Core

Graphics (GPU): NVIDIA GeForce RTX 2080

Graphics (CPU): Intel UHD Graphics 630

Hard Drive: SM961 NVMe SAMSUNG 512GB (Solid State Drive)

Motherboard: Dell 0NNNCT (C246 chipset)

Mainframe: Dell Precision 3630 Tower Desktop Mainframe

Software Versions: CUDA 11.2, torch 1.7.0, torchvision 0.8.0, python 3.8.

## 5. Discussion

### 5.1. Discussion of image segmentation results using classical machine learning methods

Six types of tumor differentiation stage data in EBHI-Seg were analyzed using classical machine learning methods to obtain the results in Table 3. Base on the Dice ratio metrics, *k*-means, MRF and Sobel show no significant differences among the three methods around 0.55. In contrast, Watershed metrics are ~0.45 on average, which is lower than the above three metrics. OTSU index is around ~0.40 because the foreground-background is blurred in some experimental samples and OTSU had a difficulty extracting a suitable segmentation threshold, which resulted in undifferentiated test results. Precision and Recall evaluation indexes for k-means, MRF, and Sobel are also around 0.60, which is higher than those for OTSU and Watershed methods by about 0.20. In these three methods, *k*-means and MRF are higher than Sobel in the visual performance of the images. Although Sobel is the same as these two methods in terms of metrics, it is difficult to distinguish foreground and background images in real images.The segmentation results for MRF are obvious but the running time for MRF is too long in comparison with other classical learning methods. Since classical machine learning methods have a rigorous theoretical foundation and simple ideas, they have been shown to perform well when used for specific problems. However, the performance of different methods varied in the present study.

### 5.2. Discussion of image segmentation results using deep learning methods

In general, deep learning models are considerably superior to classical machine learning methods, and even the lowest MedT performance is still higher than the highest accuracy of classical machine learning methods. In EBHI-Seg, the Dice ratio evaluation index of MedT reaches ~0.75. However, the MedT model size was larger and as a result the training time was too long. U-Net and Seg-Net have higher evaluation indexes than MedT, both of about 0.88. Among them, Seg-Net has the least training time and the lowest training model size. Because the normal category has fewer sample images than other categories, the evaluation metrics of the three deep learning methods in this category are significantly lower than those in other categories. The evaluation metrics of the three segmentation methods are significantly higher in the other categories, with Seg-Net averaging above 0.90 and MedT exceeding 0.80.

## 6. Conclusion and future work

The present stduy introduced a publicly available colorectal pathology image dataset containing 4456 magnified 400× pathology images of six types of tumor differentiation stages. EBHI-Seg has high segmentation accuracy as well as good robustness. In the classical machine learning approach, segmentation experiments were performed using different methods and evaluation metrics analysis was carried out utilizing segmentation results. The highest and lowest Dice ratios are 0.65 and 0.30, respectively. The highest Precision and Recall values are 0.70 and 0.90, respectively, while the lowest values are 0.50 and 0.35, respectively. All three models performed well when using the deep learning method, with the highest Dice ratio reaching above 0.95 and both Precision and Recall values reaching above 0.90. The segmentation experiments using EBHI-Seg show that this dataset effectively perform the segmentation task in each of the segmentation methods. Furthermore, there are significant differences among the segmentation evaluation metrics. Therefore, EBHI-Seg is practical and effective in performing image segmentation tasks.

## Data availability statement

The datasets presented in this study can be found in online repositories. The names of the repository/repositories and accession number(s) can be found below: EBHI-Seg is publicly available at: https://figshare.com/articles/dataset/EBHISEG/21540159/1.

## Author contributions

LS: data preparation, experiment, result analysis, and paper writing. XL: data collection and medical knowledge. WH: data collection, data preparation, and paper writing. HC: data preparation and paper writing. JC, ZF, MGa, YJ, GL, DM, ZM, QM, and DT: data preparation. HS: medical knowledge. MGr and YT: result analysis. SQ: method. CL: data collection, method, experiment, result analysis, paper writing, and proofreading. All authors contributed to the article and approved the submitted version.
